# Evaluation of ultrasonographic features of major salivary glands and oral manifestations in patients with Sjögren's Syndrome

**DOI:** 10.4317/medoral.27180

**Published:** 2025-05-27

**Authors:** Betül Taşkın, Duygu Göller-Bulut, Murat Taşçı

**Affiliations:** 1 Research Assistant; Department of Oral and Maxillofacial Radiology, Faculty of Dentistry, Bolu Abant Izzet Baysal University, Bolu, Turkey; 2 Associated Professor; Department of Oral and Maxillofacial Radiology, Faculty of Dentistry, Bolu Abant Izzet Baysal University, Bolu, Turkey; 3 Assistant Professor; Department of Rheumatology, Medical Faculty, Bolu Abant Izzet Baysal University, Bolu, Turkey

## Abstract

**Background:**

Sjögren's Syndrome (SS) is an autoimmune disease characterized by the involvement of exocrine glands and leading to various oral manifestations. The present study aimed to investigate the relationships between the salivary glands' ultrasonographic findings, the frequency of oral manifestations, the DMFT index and the unstimulated salivary flow rate in patients diagnosed with SS and to compare these parameters with healthy controls.

**Material and Methods:**

The ultrasonographic findings of the parotid and submandibular glands in 43 patients with SS were evaluated according to the Hocevar and Milic scoring system. The frequency of oral manifestations, the unstimulated salivary flow rate, and the DMFT index were calculated and the relationships between these parameters were examined. The ultrasonography findings and the DMFT index ​​of SS patients were compared with the 43 healthy control group.

**Results:**

The total Hocevar and Milic scores were higher in the patients than in the controls (*p*<0.05). The submandibular gland Hocevar score, the DMFT index, the frequency of burning mouth, and stomatitis were higher in patients with salivary flow rate ≤0.1 ml/min (*p*<0.05). Hocevar and Milic total scores were lower in patients with a salivary flow rate>0.1 ml/min. A positive correlation was observed between ultrasonography scores and DMFT in the patients (*p*<0.05). The mean total Hocevar score was found to be higher in submandibular gland than in the parotid gland (*p*=0.042).

**Conclusions:**

The increase in salivary gland ultrasonography scores in SS correlated with a decrease in unstimulated salivary flow rate, an increase in the DMFT index and some oral manifestations. Ultrasonography scores showed that, in the early stages of SS, the submandibular gland parenchyma was more affected than the parotid gland. The diagnosis of SS is difficult, and dentists can play an important role in the early diagnosis of the disease by evaluating the oral manifestations and ultrasonography findings.

** Key words:**Sjögren syndrome, salivary gland ultrasonography, oral manifestations, dmft index, salivary flow rate.

## Introduction

Sjögren syndrome (SS) is a chronic, systemic, inflammatory, autoimmune disease affecting the exocrine glands, especially the salivary and lacrimal glands, and characterized by lymphocyte infiltration and proliferation in the exocrine glands (1). Although its etiopathology is still unknown, environmental, genetic, hormonal, infectious, and psychoimmunological factors may play a role (2). SS affects 0.5-3% of the general population. It is approximately 9 times more common in women than in men and is more common around the age of 50. (3). If SS is not accompanied by another autoimmune disease, it is called primary Sjögren syndrome (pSS); if it is seen together with other autoimmune diseases (most commonly rheumatoid arthritis, systemic lupus erythematosus, and progressive systemic sclerosis), it is called secondary Sjögren syndrome (sSS) (4). Apoptosis and destruction resulting from the autoimmune response in SS is one of the main causes of hypofunction in the salivary glands (5).

Invasive salivary gland imaging modalities such as sialography and scintigraphy are being replaced by salivary gland ultrasonography (USG) in daily clinical practice in the evaluation of salivary gland involvement in SS patients (6). USG imaging, which has advantages such as being inexpensive, non-invasive, creating real-time images, not creating ionizing radiation, and being a simple and reliable technique, uses high-frequency sound waves and is widely used especially in the head and neck region. USG imaging provides detailed information about the morphological characteristics of the salivary glands and their relationships with surrounding anatomical structures. It allows the examination of the entire submandibular and sublingual glands and the superficial lobe of the parotid gland (7).

In SS, glandular involvement and loss of function in the glands result in xerostomia and xerophthalmia, which are the main symptoms of the disease and are called sicca symptoms. Xerostomia is the clinical picture responsible for the formation of oral manifestations in SS (3). Involvement of the salivary glands in these patients causes xerostomia and loss of the cleansing, lubricating, buffering, remineralizing, and antimicrobial properties of saliva (8). It was reported that individuals with xerostomia experience mastication difficulty and swallowing dry foods, problems using dentures, bad breath, a burning sensation in the mouth, changes in taste, and susceptibility to opportunistic infections such as candidiasis (9). Chronic salivary gland inflammation in patients with SS leads to loss of function and decreased salivary flow, which in turn increases the incidence of dental caries (10). This situation may result in the dental health of permanent teeth being negatively affected in SS patients. The DMFT (decayed, missing, and filled permanent teeth) index is the most widely used epidemiological scale in dentistry to determine the status of permanent teeth (11).

The aim of this study was to determine the effect of SS on the salivary glands and the parenchymal properties of the glands using USG imaging findings, to evaluate the frequency of oral manifestations in patients, to determine salivary flow rate, to calculate the DMFT index and to investigate the relationships between these parameters, and to compare the USG imaging findings and DMFT values ​​of the healthy control group and pSS patients.

## Material and Methods

-Study Population

Inclusion criteria for the study;

Patient group:

1. Patients over the age of 18,

2. Patients with USG records and patient examination records in the oral diagnosis clinic and patients diagnosed with pSS and followed up in the Rheumatology clinic of Abant Izzet Baysal University Faculty of Medicine,

3. Individuals with no systemic disease other than SS that would affect salivary secretion and the evaluated oral manifestations,

4. Individuals with no medical history (surgery, pathology, chronic disease, etc.) related to the salivary glands.

Control group:

1. Individuals matched with the patient group in terms of age and gender,

2. Individuals with panoramic radiography and USG records in the archive of the oral diagnosis clinic,

3. Individuals with no medical history (surgery, pathology, chronic disease, etc.) related to the salivary glands,

4. Individuals who do not have any systemic disease or medication use that would affect the salivary glands.

Exclusion criteria for patient and control groups;

1. Individuals under the age of 18,

2. Use of drugs and agents known to reduce saliva volume (antianxiety, anticholinergics, anticonvulsants, antidepressants, antihypertensive drugs, etc.) and increase saliva volume (artificial saliva agents, etc.),

3. Individuals with a medical history related to salivary glands (surgery, pathology, chronic diseases, etc.),

4. Patients with AIDS, HBC, HIV, cystic fibrosis, systemic lupus erythematosus, sarcoidosis, amyloidosis, head and neck radiation therapy history that affects salivary glands and causes xserostomia,

5 Individuals with any pathology that will cause a change in the echogenicity of the soft tissue surrounding the salivary glands in ultrasound images,

6. Individuals for whom the necessary data could not be obtained from the ultrasound archive and patient records.

- Preparation of data

Demographic information, gender, age, disease duration (in months), presence of oral manifestations reported in the information system (recurrent oral ulcers, xerostomia, burning mouth complaints, metallic taste in the mouth, loss of taste sensation, mastication difficulty, dysphagia, candidiasis, stomatitis) of the patients were recorded. In the statistical analysis, the patients were examined in two groups those who did not use medication and those who used immunosuppressive medication for more than 3 months. Patients using a prednisolone equivalent of less than 5 mg per day and those using hydroxychloroquine were not included in the immunosuppressive therapy group. DMFT index was calculated, and salivary flow data and parenchymal features of the submandibular and parotid glands in USG images were evaluated.

- Calculation of the DMFT Index

After the teeth were dried, detailed intraoral examinations were performed with a probe and mirror under reflector light. The DMFT index was obtained by adding the individuals' caries (D), tooth extraction due to caries (M), and filled tooth numbers (F). Individuals with congenital missing, unerupted, or supernumerary teeth and total edentulous individuals were not included.

- Measurement of Salivary Flow Rate

The salivary flow rate was assessed by measuring the amount of unstimulated saliva at rest. The resulting measurement was divided by time, and the resting salivary flow rate was recorded in milliliters/minute (ml/min) (12). The patient population was evaluated by dividing into 2 separate groups according to salivary flow rate data: ≤0.1 ml/min and >0.1 ml/min.

- USG Imaging Procedures

Bilateral submandibular and parotid salivary glands were examined with the Esaote MyLab X7 (Esaote, Genoa, Italy) device in B-mode using a 4-13 mHz high-frequency linear probe. In the evaluation of the salivary glands; patients were positioned in a supine position with their heads facing the opposite direction of the examined area. The neck was in hyperextension during the submandibular gland USG examination. The USG examination was performed by moving the probe extraorally in the transverse plane. The imaging area was adjusted to include the entire submandibular gland or a nearly entire portion of it, the widest possible parenchymal area of ​​the parotid gland, and the surrounding muscle and soft tissues.

- Evaluation of Ultrasonographic Images

**Figure 1:**
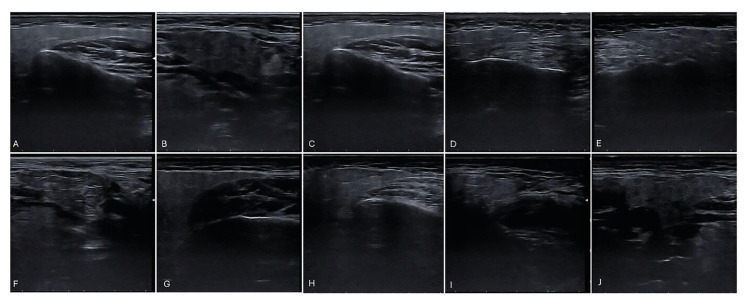
A: normal echogeneity; B: decreased echogenicity; C: homogeneous; D: mild inhomogeneity; E: evident inhomogeneity; F: grossly inhomogeneity; G: absence of hypoechoic areas; H: a few, scattered hypoechoic areas; I: several hypoechoic areas; J: numerous hypoechoic areas.

USG images were examined according to the Hocevar scoring system and coded as follows (13):

The parenchymal echogenicity of the salivary gland; 0: echogenicity was comparable to the surrounding anatomical structures (muscle and subcutaneous fat tissue), 1: decreased echogenicity (Fig. 1).

Parenchymal homogeneity; 0: homogeneous, 1: mild inhomogeneity, 2: evident inhomogeneity, 3: grossly inhomogeneity (Fig. 1).

Presence of hypoechoic areas; 0: absent, 1: a few, scattered, 2: several, 3: numerous (Fig. 1).

**Figure 2:**
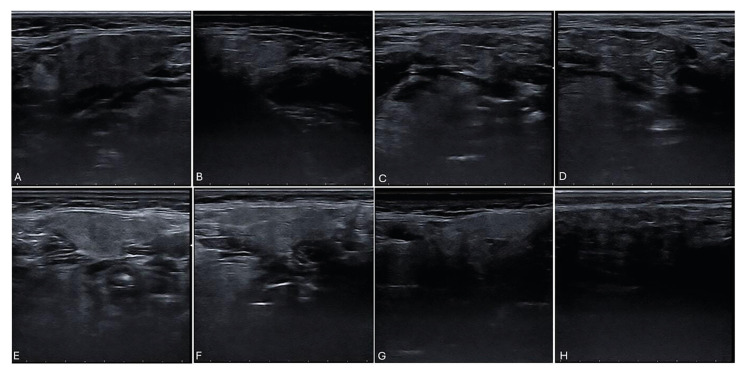
A: absence of hyperechoic reflections; B: a few, scattered hyperechoic reflections; C: several hyperechoic reflections; D: numerous hyperechoic reflections; E: clear, well defined borders; F: slightly less defined borders; G: ill-defined borders; H: unidentifiable, borders.

Presence of hyperechoic reflections; 0: absent, 1: a few, scattered, 2: several, 3: numerous (Fig. 2).

Clearness of glandular borders: 0: clear, well-defined borders; 1: slightly less defined borders; 2: ill-defined borders; 3: unidentifiable, borders indistinguishable from surrounding parenchyma (Fig. 2).

Scores were evaluated in separate groups for the right and left parotid and submandibular glands. The higher score from the scores obtained from the right and left sides for the parotid and submandibular glands was accepted and the total gland score was obtained. Finally, the total Hocevar score was calculated by summing the degrees of the five parameters described above for all four glands.

To compare salivary gland parenchymal homogeneity with other data, parotid and submandibular gland parenchymal homogeneity scores were collected for each patient and a score between 0 and 12 was obtained, similar to the Milic Score (14).

- Statistical Analysis

Descriptive statistics of the measurements were calculated as mean, standard deviation (SD), minimum and maximum, number, and % frequencies. The Shapiro-Wilks test was used to examine the compliance of the data obtained with the normal distribution. Independent samples t-test was used to compare various subgroups in terms of numerical features showing normal distribution, and Mann-Whitney U test was applied for non-normally distributed variables Additionally, correlations between numerical variables were evaluated using Pearson correlation analysis. Relationships between categorical variables were analyzed using the Pearson chi-square test. A *p-value* of ≤0.05 was considered statistically significant. All analyses were performed using SPSS software (version 23).

## Results

A total of 86 individuals were included in the study, 43 SS patients and 43 healthy controls. The study population included 4 male patients, 2 (4.7%) in the patient group and 2 (4.7%) in the control group. The mean age of the patient group was 50.09±11.235 and the mean age of the control group was 46.3±9.033, and no significant difference was found between the age distribution of the groups (*p*=0.088).

According to the data obtained from the records of the Rheumatology Clinic of the Faculty of Medicine; Anti SS-A (74.42%) and ANA (antinuclear antibody) (86.05%) were positive in the majority of the patients. The rate of patients with salivary flow rate ≤ 0.1 ml/min was found to be 55.8%. According to the scoring reported by Chisholm-Mason (15), minor salivary gland biopsy results were 17 (39.54%) patients with stage 0, 10 (23.25%) patients with stage 1, 9 (20.93%) patients with stage 2, 3 (6.98%) patients with stage 3 and 4 (9.30%) patients with stage 4. The number of patients with a positive Schirmer test result (≤5 mm in at least one eye) was 22 (51.16%).

**Figure 3:**
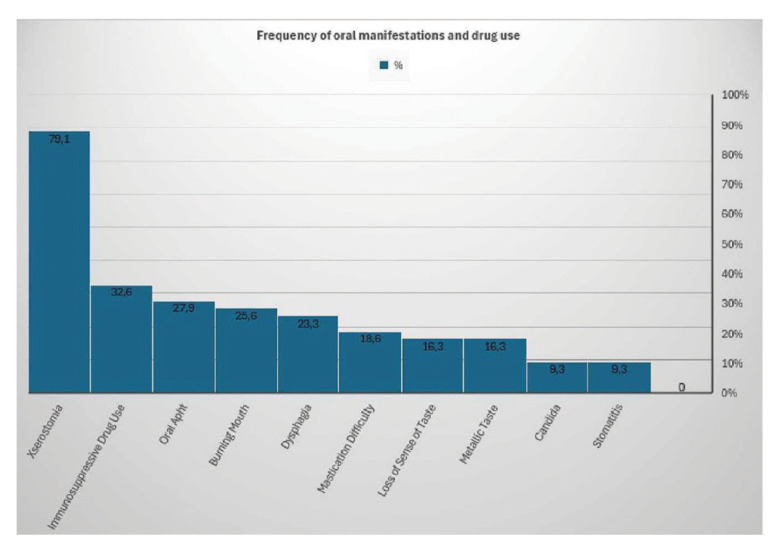
Data on frequency of medication use and the oral manifestations in the patient group.

Fig. 3 shows the distribution of responses given by the patient group to categorical questions obtained from anamnesis records. The frequency of immunosuppressive drug use in patients was 32.6%. The frequency of xerostomia was 79.1%, burning mouth was 25.6%, oral aphth was 27.9%, candidiasis was 9.3%, stomatitis was 9.3%, loss of taste was 16.3%, the metallic taste was 16.3%, mastication difficulty was 18.6% and dysphagia was 23.3%.

The frequency of mastication difficulty and dysphagia was significantly higher in those who did not use immunosuppressive drugs and was not observed in those who used drugs (*p* =0.029 and *p* =0.012, respectively). The mean disease duration was found to be significantly higher in patients with stomatitis, loss of taste sensation, and mastication difficulty (*p*=0.039, *p*=0.022, and *p*=0.005; respectively) (Table 1).

The mean DMFT index was 16.62±5.941 in the patient group and 14.98±6.952 in the control group, and there was no significant difference between the two groups in terms of DMFT (*p* =0.264; respectively). The mean disease duration was 37.14±51.805 months. The relationship between age disease duration and DMFT in the patient group was not found to be significant (*p*>0.05). The relationships between salivary flow rate, DMFT, and oral symptoms in patients are given in Table 2. The frequency of burning mouth and stomatitis was found to be significantly higher in those with salivary flow rate ≤0.1 ml/min (*p* =0.044 and *p* =0.026; respectively). In addition, the mean DMFT was found to be significantly higher in those with salivary flow rate ≤0.1 ml/min (*p* =0.001). No significant relationship was found between DMFT and the frequency of oral manifestations (*p*>0.05).

The mean parotid gland Hocevar score was 9.81±3.088 and the mean submandibular gland Hocevar score was 11.51±3.089. The mean Hocevar total score in control group was 4.42±2.566 and in patient group was 21.25±4.990. The mean Milic total score in control group was 1.72±1.161 and in patient group was 6.16±1.772. The mean Hocevar total score and Milic total score were found to be significantly higher in patients (*p* =0.001 and *p* =0.001, respectively).

The relationships between the parotid gland Hocevar score, submandibular gland Hocevar score, and salivary flow rate and oral symptoms in patients are given in Table 3. The mean submandibular gland Hocevar score was found to be significantly lower in those with salivary flow rate>0.1 ml/min (*p* =0.002) and significantly higher in those with burning mouth (*p* =0.027).

The relationships between Hocevar total score and Milic total score and immunosuppressive drug use status, salivary flow rate, and oral symptoms in patients are given in Table 4. The mean Hocevar total score and Milic total score were found to be significantly higher in individuals using immunosuppressive drugs (*p*=0.020, *p* =0.010; respectively). In addition, the mean Hocevar total score and Milic total score were found to be significantly lower in those with salivary flow rate>0.1 ml/min (*p* =0.03 and *p* =0.021; respectively). No significant relationship was found between oral symptoms and Hocevar total score and Milic total score.

**Figure 4:**
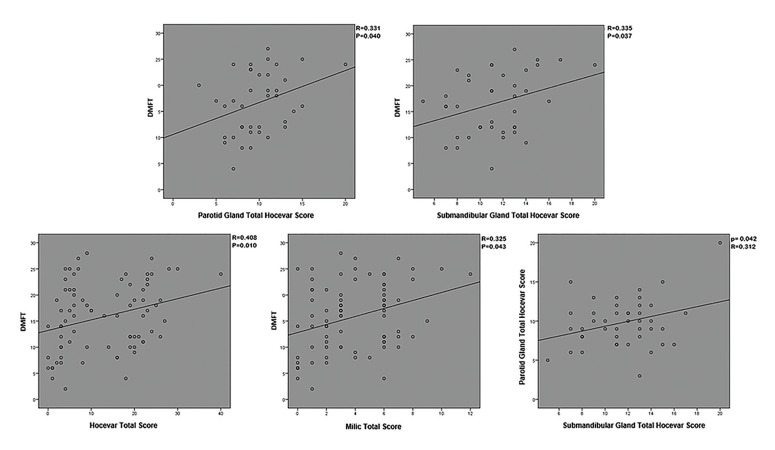
Correlations between DMFT and USG scores, and between the total scores of the parotid and submandibular glands.

There was a significant positive correlation between the Hocevar score, Hocevar total score Milic total score, and the DMFT index in the patient group (*p*<0.05). In addition, there was a significant positive correlation between the parotid gland Hocevar score and the submandibular gland Hocevar score in the patient group (*p*=0.042; R=0.312). In addition, the mean submandibular gland Total Hocevar score was found to be higher than the parotid gland total score (Fig. 4).

## Discussion

SS is a chronic, inflammatory, autoimmune and lymphoproliferative disease characterized by mononuclear cell infiltration of exocrine glands and is difficult to diagnose (16). In the presented study, the patient group consists of patients who were diagnosed with SS by evaluating the diagnostic criteria of the Rheumatology clinic. It has been reported in the literature that SS is seen 9 times more in females than in males and that the incidence is highest in the fourth and fifth decades of life. (17). In the present study, there was a significant dominance in the female population (41; 95.3%) compared to males (2, 4.7%) in the patient group. There was no significant difference between the groups in terms of age and gender. This allowed the comparison of USG findings and DMFT scores between the groups, independent of age and gender.

Crincoli *et al*. (18) reported that 91.7% of 72 SS patients had at least one oral finding. The frequency of xerostomia in SS patients was reported as 91.67%, taste disturbances as 31.94%, dysphagia as 14%, candidiasis as 12.5%, and stomatitis as 12.5%. In the study conducted by Marton *et al*. (19) on 49 SS patients, xerostomia was reported as 92%, dysphagia as 34%) candidiasis as 34%, and stomatitis as 18%. In the present study, the frequency of xerostomia in the SS group was found to be 79.1%, burning mouth 25.6%, oral aphtha 27.9%, candidiasis 9.3%, stomatitis 9.3%, loss of taste 16.3%, metallic taste 16.3%, mastication difficulty 18.6% and dysphagia 23.3%. We think that the relatively lower frequency of oral symptoms in this study compared to other studies may be due to the lower average age and mean disease duration of our patient group, the fact that most of our patient group was in the early stages of the disease and the data on symptoms were subjective.

In the present study, 32.6% of the patient group was using immunosuppressive drugs and, similar to the previous study (20) conducted on SS patients, no significant relationship was found between unstimulated salivary flow rate and immunosuppressive drug use. However, mastication difficulty and dysphagia were found more frequently in patients not using immunosuppressive drugs. These results may be due to the subjective nature of oral manifestations and the possibility of a placebo effect of immunosuppressive use on patients.

Theander *et al*. (21) followed SS patients for 5 years and reported that dry eyes, dry mouth, and quality of life were generally quite sTable. Age and disease duration were not correlated with any objective oral measurements at baseline or follow-up. It has been suggested that this may be because patients have developed effective coping strategies (21). In the present study, the mean disease duration was significantly higher in those with stomatitis, loss of taste, and mastication difficulty. As we stated in the present study, the frequency of oral symptoms was lower than the literature data. When the findings were evaluated, the mean disease duration was more than 5 years in those with stomatitis, loss of taste, and mastication difficulty symptoms. Since patient follow-up was not performed due to the retrospective design of the present study, it was not possible to evaluate how the symptoms changed compared to the beginning or the advanced duration of the disease.

It has been reported that the feeling of xerostomia experienced by SS patients is due to the decrease in total salivary volume (22). Marton *et al*. (19) reported an unstimulated salivary flow rate of ≤0.1 ml/min in 33 of 49 (67%) SS patients. Serrano *et al*. (23) found that 60% of 61 patients diagnosed with SS had unstimulated salivary flow rate ≤0.1 ml/min and found a negative correlation between the severity of dry mouth and salivary flow rate in these patients. Similarly in the study of Osailan *et al*. (24) a negative correlation was found between clinical dry mouth severity and unstimulated salivary flow rate in SS. In the present study, the frequency of burning mouth and stomatitis was significantly higher in those with a salivary flow rate of ≤0.1 ml/min. No significant correlation was found between other oral manifestations and salivary flow rate. These findings are subjective because they are based on patient reports, so it is an expected result that the results between studies will differ.

In the literature, DMFT values ​​were found to be higher in SS patients compared to healthy controls (25). In the study of Glavina *et al*. (26) comparing the DMFT index in 31 pSS and 28 control patients, no statistically significant difference was observed between the groups and it was suggested that this might be due to the small sample size. In the study by Molania *et al*. (27) comparing 45 SS patients and 45 healthy controls, no significant difference was found between the groups in terms of DMFT index, and also no significant relationship was observed between DMFT and oral health-related quality of life questionnaire results. In the present study, similar to previous studies (26,27), no statistically significant difference was found between the patient and control groups in terms of DMFT index. The patients in the control group applied to our clinic with various existing dental complaints, and the majority of our patient group consisted of patients with a diagnosis period of less than 1 year, and the tooth brushing information in the anamnesis records of the groups was similar. We think that these factors may be the reason why the DMFT values ​​did not show significant differences between groups.

There is no specific method with confirmed diagnostic validity to demonstrate salivary gland involvement and function in the diagnosis of SS, and accepted methods such as salivary scintigraphy, parotid sialography, and unstimulated salivary flow rate have limited sensitivity and specificity (13). Unstimulated salivary flow rate is clinically applicable, easy and simple, highly specific for xerostomia, and is becoming an important diagnostic tool together with salivary gland ultrasonography (28). Ferguson *et al*. (29) reported that low salivary flow rates were associated with high rates of dental caries in SS patients. Similarly, in the present study, the mean DMFT was found to be significantly higher in those with unstimulated salivary flow rates of ≤0.1 ml/min. In contrast, Bookman *et al*. (30) reported no significant correlation between unstimulated salivary flow and DMFT scores and dry mouth duration in 197 pSS patients. When the literature was reviewed, no other study was found evaluating the correlation between DMFT and salivary flow rates in SS patients, and therefore, these parameters could not be compared and discussed.

In the literature, it has been emphasized that salivary gland USG features are important in the diagnosis of SS (31,32). It is difficult to reach a consensus on the most appropriate diagnosis and scoring method for USG abnormalities in individuals with SS (33). Hocevar (13) reported that the USG scoring system proposed has a high diagnostic value. Therefore, in the present study, Hocevar (13) score, which evaluates echogenicity, parenchymal homogeneity, hypoechoic area, hyperechoic reflection, glandular border parameters was used, and the Milic (14) score, which evaluates parenchymal homogeneity was used. Niemela *et al*. (34) found that the scores of parenchymal structure in USG of parotid and submandibular glands in pSS patients were related to each other. In the present study, a positive correlation was found between the parotid gland Hocevar score and the submandibular gland Hocevar score in the patient group. In the literature, USG scores of patients with SS and sicca symptoms were found to be higher in the patient group than in the control group (35). Similarly, in the present study, it was observed that the Hocevar total score and Milic total score averages were significantly higher in the patients than in the control group.

Lee *et al*. (35) found lower unstimulated salivary flow rate in the positive USG group (those with Hocevar USG score ≥14) in patients with pSS and sicca symptoms and reported a relationship between unstimulated salivary flow rate and USG scores. Baldini *et al*. (36) found that the USG score correlated with unstimulated salivary flow in the early diagnosis of pSS. Yalçınkaya *et al*. (37) performed USG examinations according to Hocevar and Milic scores in 66 SS patients and found higher Hocevar and Milic scores in 21 SS patients with reduced unstimulated salivary flow rate. In addition, severe parotid gland involvement was associated with low salivary flow rate and poor oral health and quality of life. Similarly, in the present study, the mean Hocevar total score, Milic total score, and submandibular gland Hocevar score were found to be significantly lower in those with salivary flow rate>0.1 ml/min. As a result of these findings, it was confirmed that there was a relationship between salivary flow rate and salivary gland involvement in USG.

Niemela *et al*. (34) found no relationship between USG features and disease duration in pSS patients. In the study by Theander *et al*. (38) evaluating the salivary gland parenchymal homogeneity as defined by Hocevar in pSS patients and sicca control groups, age, disease duration, and dry mouth complaints did not show any difference between normal and abnormal USG. Zabotti *et al*. (39) evaluated 75 pSS patients and reported a relationship between hyperechoic areas in USG examination and dry mouth severity determined by visual analog scale (VAS) and unstimulated salivary flow rate. Lee *et al*. (35) found no significant difference in the positive USG group in terms of age, disease duration, gender, and dryness symptoms. Similarly, in the present study, no significant relationship was observed between disease duration and oral symptoms and USG scores, no relationship was found between the mean parotid gland Hocevar score and salivary flow rate and oral symptoms, but there was a correlation between the increased in submandibular gland Hocevar score and burning mouth. Additionally, similar to studies in the literature (14,34), no significant relationship was observed between total USG scores and age.

In the study by Delli *et al*. (40), the Hocevar score of the submandibular gland was found to be higher than the parotid gland. Pijpe *et al*. (20) found that the reduction in unstimulated salivary flow rate in the submandibular gland was more pronounced compared to the parotid gland in patients with a short disease duration (less than 1 year) than in those with a long disease duration. These findings suggest that during the disease process, signs of hyposalivation are first observed in the submandibular gland which is responsible for most of the salivary secretion. In our study, similar to Delli *et al*. (40), those with partially defined borders were found more in the parotid gland, and those with poorly defined borders were found more in the submandibular gland, and the total homogeneity scores in the submandibular gland were higher than in the parotid gland. In addition, the mean Hocevar score in the submandibular gland was higher than in the parotid gland. Functional changes in Sjögren's Syndrome (SS) are observed earlier and more prominently in the submandibular gland compared to the parotid gland (20). Similarly, in this study, it is thought that the submandibular salivary gland is more affected than the parotid gland, likely due to the majority of patients having a short disease duration, consistent with previous findings.

Yalçınkaya *et al*. (37) conducted a USG examination on 66 SS patients using the Hocevar and Milic scoring systems and evaluated the Oral Health Impact Profile (OHIP-14), Oral Health-Related Quality of Life (OHRQoL), various periodontal and gingival indices, the presence of dental caries, and the frequency of natural tooth count. No significant association was found between USG scores and the number of teeth or dental caries in patients with pSS (37). In our study, a significant positive correlation was observed between the parotid gland Hocevar score, submandibular gland Hocevar score, total Hocevar score, and total Milic score with the DMFT index in the patient group. A comparison could not be made since there was no study comparing similar parameters in the literature. Increased USG scores have been suggested in the literature to be an indicator of gland involvement. As gland involvement increases, salivary flow decreases and the positive effects of saliva on oral flora and tissues decrease, increasing the frequency of dental problems and explaining the positive correlation between USG scores and DMFT.

## Conclusions

In the present study, significant relationships were found between USG scores and the frequency of some oral manifestations, and the mean USG score was higher in those with salivary flow rate ≤0.1 ml/min. There was also a significant positive correlation between USG scores and the DMFT index. In the early stages of SS, the submandibular gland parenchyma is more affected than the parotid gland, and the increase in the DMFT index and the frequency of oral manifestations and the decrease in salivary flow rate are thought to be the result of this. New prospective studies are needed in a larger population, where oral manifestations can be evaluated objectively and patients are followed up at certain intervals. The role of dentists in early diagnosis and treatment of SS patients is important. The collaboration of dentists and rheumatologists in the diagnosis and treatment of patients helps in the early diagnosis and rehabilitation of the SS.

## Figures and Tables

**Table 1:** Relationships between the parameters evaluated with the use of immunosuppressive drugs and disease duration in patients.

		Immunosuppressive Drug Use						Disease Duration			
		No		Yes		Total	p	N	Mean	SD	p
		N	%	N	%	N					
Salivary Flow Rate	≤0.1	16	55.2	8	57.1	24	0.903	24	45.33	60.762	0.223
	>0.1	13	44.8	6	42.9	19		19	26.79	36.633	
Xserostomia	Absent	6	20.7	3	21.4	9	0.955	9	35.33	33.223	0.908
	Present	23	79.3	11	78.6	34		34	37.62	56.098	
Burning Mouth	Absent	22	75.9	10	71.4	32	0.755	32	31.25	44.585	0.207
	Present	7	24.1	4	28.6	11		11	54.27	68.378	
Oral Apht	Absent	21	72.4	10	71.4	31	0.946	31	36.52	51.154	0.901
	Present	8	27.6	4	28.6	12		12	38.75	55.737	
Candida	Absent	26	89.7	13	92.9	39	0.735	39	35.72	52.293	0.580
	Present	3	10.3	1	7.1	4		4	51.00	51.498	
Stomatitis	Absent	25	86.2	14	100.0	39	0.145	39	26.18	35.631	0.039*
	Present	4	13.8	0	0.0	4		4	144.00	68.586	
Loss of Sense of Taste	Absent	23	79.3	13	92.9	36	0.260	36	29.25	42.819	0.022*
	Present	6	20.7	1	7.1	7		7	77.71	76.135	
Metallic Taste	Absent	25	86.2	11	78.6	36	0.525	36	35.03	52.749	0.551
	Present	4	13.8	3	21.4	7		7	48.00	48.898	
Mastication Difficulty	Absent	21	72.4	14	100.0	35	0.029	35	26.77	36.791	0.005*
	Present	8	27.6	0	0.0	8		8	82.50	81.479	
Dysphagia	Absent	19	65.5	14	100.0	33	0.012*	33	34.39	49.549	0.534
	Present	10	34.5	0	0.0	10		10	46.20	60.630	
Immunosuppressive Drug Use	Absent	-						29	37.07	57.126	0.990
	Present	-						14	37.29	40.519	

N: number, %: percentage, SD: standart deviation, * Statistically significant (p ≤0.05).

**Table 2:** Relationships between salivary flow rate and oral symptoms in patients.

		Salivary Flow Rate						DMFT			
		≤ 0.1		>0.1		Total									
		N	%	N	%	N	p	N	Mean	SD	p
Xserostomia	Absent	3	12.5	6	31.6	9	0.127	7	15.86	5.273	0.715
	Present	21	87.5	13	68.4	34		32	16.78	6.142	
Burning Mouth	Absent	15	62.5	17	89.5	32	0.044*	30	15.97	5.696	0.218
	Present	9	37.5	2	10.5	11		9	18.78	6.572	
Oral Apht	Absent	16	66.7	15	78.9	31	0.373	28	17.11	6.244	0.417
	Present	8	33.3	4	21.1	12		11	15.36	5.143	
Candida	Absent	21	87.5	18	94.7	39	0.417	35	16.54	5.997	0.825
	Present	3	12.5	1	5.3	4		4	17.25	6.238	
Stomatitis	Absent	20	83.3	19	100.0	39	0.026*	35	16.17	5.803	0.171
	Present	4	16.7	0	0.0	4		4	20.50	6.557	
Loss of Sense of Taste	Absent	18	75.0	18	94.7	36	0.082	33	16.12	5.957	0.228
	Present	6	25.0	1	5.3	7		6	19.33	5.538	
Metallic Taste	Absent	19	79.2	17	89.5	36	0.363	33	16.67	6.213	0.901
	Present	5	20.8	2	10.5	7		6	16.33	4.590	
Mastication Difficulty	Absent	19	79.2	16	84.2	35	0.673	32	16.53	5.483	0.853
	Present	5	20.8	3	15.8	8		7	17.00	8.246	
Dysphagia	Absent	17	70.8	16	84.2	33	0.302	31	16.61	5.766	0.996
	Present	7	29.2	3	15.8	10		8	16.63	7.009	
Immunosuppressive Drug Use	Absent	-						26	16.35	6.203	0.695
	Present	-						13	17.15	5.580	
Salivary Flow Rate	≤0.1	-						20	19.50	5.155	0.001*
	>0.1	-						19	13.58	5.242	

N: number, %: percentage, SD: standart deviation, * Statistically significant (p ≤0.05).

**Table 3:** Relationships between Hocevar scores of parotid and submandibular glands and salivary flow rate and oral symptoms in patients.

		Hocevar Score (Parotid Gland)				Hocevar Score (Submandibular Gland)			
		N	Mean	SD	p	N	Mean	SD	p
Salivary Flow Rate	≤0.1	24	10.54	3.189	0.082	24	12.75	2.923	0.002*
	>0.1	19	8.89	2.767		19	9.95	2.592	
Xserostomia	Absent	9	8.78	2.333	0.263	9	11.44	2.506	0.943
	Present	34	10.09	3.232		34	11.53	3.259	
Burning Mouth	Absent	32	9.81	3.402	0.996	32	10.91	3.009	0.027*
	Present	11	9.82	2.040		11	13.27	2.724	
Oral Apht	Absent	31	9.84	3.247	0.934	31	11.48	3.182	0.926
	Present	12	9.75	2.768		12	11.58	2.968	
Candida	Absent	39	9.97	3.141	0.293	39	11.26	3.076	0.091
	Present	4	8.25	2.217		4	14.00	2.160	
Stomatitis	Absent	39	9.77	3.232	0.771	39	11.44	3.177	0.622
	Present	4	10.25	0.957		4	12.25	2.217	
Loss of Sense of Taste	Absent	36	9.58	3.255	0.272	36	11.31	3.267	0.327
	Present	7	11.00	1.732		7	12.57	1.718	
Metallic Taste	Absent	36	9.56	3.084	0.217	36	11.42	3.157	0.653
	Present	7	11.14	2.968		7	12.00	2.887	
Mastication Difficulty	Absent	35	9.80	3.376	0.921	35	11.49	3.329	0.910
	Present	8	9.88	1.356		8	11.63	1.847	
Dysphagia	Absent	33	9.88	3.426	0.727	33	11.48	3.318	0.919
	Present	10	9.60	1.647		10	11.60	2.319	

N:number, %: percentage, SD: standart deviation, * Statistically significant (p ≤0.05).

**Table 4:** Relationships between Hocevar total score and Milic total score and medication use, salivary flow rate and oral symptoms in patients.

		Hocevar Score (Total)				Milic Score (Total)			
		N	Mean	SD	p	N	Mean	SD	p
Immunosuppressive Drug Use	Absent	29	20.14	5.350	0.020*	29	5.69	1.795	0.010*
	Present	14	23.86	2.958		14	7.14	1.292	
Salivary Flow Rate	≤0.1	24	23.29	4.704	0.003*	24	6.71	1.601	0.021*
	>0.1	19	18.89	4.293		19	5.47	1.775	
Xserostomia	Absent	9	20.22	3.346	0.453	9	5.78	1.202	0.470
	Present	34	21.65	5.342		34	6.26	1.896	
Burning Mouth	Absent	32	20.75	5.406	0.183	32	6.00	1.918	0.310
	Present	11	23.09	3.081		11	6.64	1.206	
Oral Apht	Absent	31	21.35	5.695	0.990	31	6.29	1.987	0.455
	Present	12	21.33	2.570		12	5.83	1.030	
Candida	Absent	39	21.26	5.215	0.709	39	6.23	1.828	0.439
	Present	4	22.25	1.708		4	5.50	1.000	
Stomatitis	Absent	39	21.23	5.204	0.634	39	6.15	1.857	0.919
	Present	4	22.50	1.915		4	6.25	0.500	
Loss of Sense of Taste	Absent	36	20.92	5.245	0.201	36	6.06	1.866	0.374
	Present	7	23.57	2.637		7	6.71	1.113	
Metallic Taste	Absent	36	21.00	5.275	0.304	36	6.06	1.851	0.374
	Present	7	23.14	2.734		7	6.71	1.254	
Mastication Difficulty	Absent	35	21.31	5.449	0.926	35	6.17	1.963	0.948
	Present	8	21.50	2.268		8	6.13	0.354	
Dysphagia	Absent	33	21.39	5.545	0.916	33	6.27	1.989	0.466
	Present	10	21.20	2.616		10	5.80	0.632	

N: number, %: percentage, SD: standart deviation, * Statistically significant (p≤0.05).
